# *Musa balbisiana* Fruit Rich in Polyphenols Attenuates Isoproterenol-Induced Cardiac Hypertrophy in Rats via Inhibition of Inflammation and Oxidative Stress

**DOI:** 10.1155/2020/7147498

**Published:** 2020-01-27

**Authors:** Sima Kumari, Parmeshwar B. Katare, R. Elancheran, Hina L. Nizami, Bugga Paramesha, Sudheer Arava, Partha Pratim Sarma, Roshan Kumar, Dinesh Mahajan, Yashwant Kumar, Rajlakshmi Devi, Sanjay K. Banerjee

**Affiliations:** ^1^Translational Health Science and Technology Institute (THSTI), Faridabad, India; ^2^Drug Discovery Lab, Department of Chemistry, Annamalai University, Tamil Nadu, India; ^3^Department of Pathology, All India Institute of Medical Sciences (AIIMS), New Delhi, India; ^4^Institute of Advanced Study in Science and Technology, Guwahati, 781035 Assam, India

## Abstract

*Musa balbisiana* Colla (Family: Musaceae), commonly known as banana and native to India and other parts of Asia, is very rich in nutritional value and has strong antioxidant potential. In the present study, we have developed *Musa balbisiana* (MB) fruit pulp powder and evaluated its cardioprotective effect in cardiac hypertrophy, which is often associated with inflammation and oxidative stress. An ultra-high-pressure liquid chromatography-mass spectrometer (UPLC-MS/MS) has been used for the detection and systematic characterization of the phenolic compounds present in *Musa balbisiana* fruit pulp. The cardioprotective effect of MB was evaluated in a rat model of isoproterenol- (ISO-) induced cardiac hypertrophy by subcutaneous administration of isoproterenol (5 mg/kg^−1^/day^−1^), delivered through an alzet minipump for 14 days. Oral administration of MB fruit pulp powder (200 mg/kg/day) significantly (*p* < 0.001) decreased heart weight/tail length ratio and cardiac hypertrophy markers like ANP, BNP, *β*-MHC, and collagen-1 gene expression. MB also attenuated ISO-induced cardiac inflammation and oxidative stress. The *in vivo* data were further confirmed *in vitro* in H9c2 cells where the antihypertrophic and anti-inflammatory effect of the aqueous extract of MB was observed in the presence of ISO and lipopolysaccharide (LPS), respectively. This study strongly suggests that supplementation of dried *Musa balbisiana* fruit powder can be useful for the prevention of cardiac hypertrophy via the inhibition of inflammation and oxidative stress.

## 1. Introduction

Cardiac hypertrophy leading to heart failure is one of the major causes of morbidity and mortality in the world. It is characterized by an increase in cardiac muscle mass and accumulation of myocardial scarring (collagen) with a decrease in its pumping ability. Cardiac hypertrophy is typically characterized by an enlargement of the heart and an increase in myocyte cell volume, and caused by various factors including hemodynamic stress [1]. Oxidative stress and inflammation play a crucial role in the development of various diseases, including cardiovascular disease, diabetes, obesity, cancer, metabolic syndrome, and other chronic diseases. Several reports in scientific literature indicate that oxidative stress induced by reactive oxygen species (ROS) plays a significant role in activating stress-sensitive signaling pathways that regulate gene expression leading to cellular damage [2–4]. Therefore, finding novel strategies and therapeutic interventions to prevent chronic diseases like cardiovascular disease, diabetes, and obesity via the inhibition of oxidative stress and inflammation are very important. Cardiac hypertrophy is a progressive and chronic disease and can be modified by nutritional and other lifestyle modifications. Currently, most of the heart failure patients are treated with different existing drugs with limited success. Hence, our focus is to develop a nutritional agent from natural resources available from North-east India to prevent cardiac hypertrophy and heart failure.

*Musa balbisiana* Colla (Family: Musaceae), commonly known as banana, is native to India and other parts of Asia and has been utilized in folk medicine since a long time by the tribal people of North-east India [5]. However, literature reviews have indicated that different banana species are utilized traditionally for the treatment of cardiac diseases, diabetes, inflammation, hypertension, diarrhoea, and dysentery [6, 7]. The root, stem juice, and ash of banana leaves are also utilized in venereal diseases and blood disorders and as an anthelmintic [5–7]. The World Health Organization (WHO) believes that a significant percentage of the population of developing countries rely on natural resources for their primary health care needs. Recently, many research findings have shown that the consumption of polyphenols or polyphenol-rich foods has an inverse relationship to the manifestation of disease state [8–10]. Moreover, it was established that dietary phenolic compounds such as chlorogenic acid, quercetin and apigenin have been associated with reducing the risk of both chronic and acute diseases [11, 12]. These therapeutic responses to polyphenols have been attributed to their antioxidant and anti-inflammatory properties [12]. In recent literature, the role of *M. balbisiana* root extract in reducing hyperlipidemia and diabetes has been well established [5]. However, chemical characterization, as well as the role of *M. balbisiana* fruit pulp to improve cardiac health through modulation of oxidative stress and inflammation, was not reported yet.

In the present study, we have developed a *Musa balbisiana* fruit pulp powder and characterised its phenolic compounds by UPLC/MSMS. The powder was tested in a rat model of cardiac hypertrophy by the administration of isoproterenol (5 mg/kg^−1^/day^−1^) through an alzet minipump for 14 days. Our data has clearly indicated that cardiac hypertrophy was associated with myocardial inflammation and oxidative stress which were attenuated after oral administration of *Musa balbisiana* (200 mg/kg/day) fruit powder.

## 2. Materials and Methods

### 2.1. Dosage Information

*Musa balbisiana* fruits were purchased from a local market of Guwahati, Kamrup district of Assam (situated in between 25°43′-26°53′ north latitude and 90°39′-92°11′ east latitude) in the months of March to September 2018. The process of producing banana powder included the steps of collecting banana (*Musa balbisiana*), washing, and chopping, additionally grounding and drying them for 2 to 4 days in an oven at temperatures of 40 to 80°C until they were crisped properly. Rats were fed with fresh aqueous suspension of the powder every day.

### 2.2. Systematic Characterization and Identification of the Bioactive Compound

*Musa balbisiana* fruit pulp powder extract was prepared by homogenizing 30 mg of MB powder in 300 *μ*L of water and centrifuging at 15,000 rpm for 15 min at 4°C. The resulting supernatant was stored at -80°C for further analysis. Extracted metabolites were dried in a SpeedVac vacuum concentrator and stored at -80°C till use. Samples were reconstituted in 15% methanol before LC-MS analysis [8]. All data were acquired on the Orbitrap Fusion Mass Spectrometer equipped with a heated electrospray ionization (HESI) source. Data were acquired on positive and negative modes at 120,000 resolution in the MS1 mode and 30,000 resolution in the data-dependent MS2 scan mode. We used a spray voltage of 4000 and 35,000 volts for positive and negative modes, respectively. Sheath gas and auxiliary gas were set to 42 and 11, respectively. Mass scan range was 50-1000 *m*/*z*. We used an automatic gain control target at 200,000 ions and maximum injection time at 80 ms for MS and an automatic gain control target at 20,000 ions and maximum injection time at 60 ms for MSMS. The aqueous extract of *Musa balbisiana* was separated on a “UPLC Ultimate 3000” using an HSS T3 column (100 × 2.1 mm i.d., 1.7 micrometers, waters) maintained at 40 degrees C temperature. The mobile phase A was water with 0.1% formic acid, and mobile phase B was acetonitrile with 0.1% formic acid. The elution gradient is used as follows: 0 min, 1% B; 1 min, 15% B; 4 min, 35% B; 7 min, 95% B; 9 min, 95% B; 10 min, 1% B; and 14 min, 1% B. The flow rate of 0.3 mL/min and sample injection volume was 5 microliters. All acquired data has been processed using Progenesis QI software using the default setting. The untargeted metabolomics workflow of Progenesis QI was used to perform retention time alignment, feature detection, and elemental composition prediction. Identification of metabolites has been done on the basis of an in-house metabolite with accurate mass, fragmentation pattern, and retention time information. We have also used spectral data matching with mzCloud and MassBank and Global Natural Products Social Molecular Networking (GNPS) curated spectral libraries of natural products for the fragmentation match for the identification of metabolites. Confirmation of the identity of metabolites has been further verified through running two authentic standards of respective metabolites obtained from the MS/MS fragmentation match. We have matched retention time and the MS/MS fragmentation pattern of the standard to confirm the identity.

### 2.3. Animals

All animal experiments were undertaken with the approval of the Institutional Animal Ethics Committee of the Translational Health Science and Technology Institute, Faridabad (IAEC/THSTI/2017-2019). Male Sprague-Dawley rats (250 g) were obtained from the National Institute of Pharmaceutical Education and Research (NIPER), SAS Nagar, India. The animals were housed in the Small Animal Facility of Translational Health Science and Technology Institute, Faridabad. The animal house was maintained at a temperature of 22 ± 2°C with a relative humidity of 40 ± 15% and 12 h dark/light cycle throughout the study. Rats had free access to food and water *ad libitum*.

### 2.4. Induction of Cardiac Hypertrophy in Rats and Treatment Schedule

Weight-matched (180-200 g) male Sprague-Dawley rats were utilized for this study. Control rats were fed with normal saline daily for 14 days. Cardiac hypertrophy in rats was developed by the administration of isoproterenol by placing the alzet pumps subcutaneously for 14 days at a dose of 5 mg/kg/day. Animals were anesthetized using a mixture of ketamine (75 mg/kg, IP) and xylazine (5 mg/kg, IP) for the surgical insertion of the alzet pump. *Musa balbisiana* fruit powder in aqueous suspension form was fed to the rats of the cardiac hypertrophy group by oral gavage every day at a fixed time (10:00 a.m.) for 14 days at a dose of 200 mg/kg/day (*N* = 10). Body weight gain and tail length were monitored during the study period. At the end of the study, all animals were sacrificed with a high dose of anesthesia; hearts were collected and stored in -80°C or 4% formalin for downstream analysis.

### 2.5. Cardiac Hypertrophy Parameter

Isoproterenol through the minipump at a dose of 5 mg/kg/day body weight for 14 days causes cardiac hypertrophy in rats [13]. In each group, the heart weight/tail length ratio was measured on the day of sacrifice. Heart weight was measured after rinsing freshly excised heart in ice-cold PBS and drying on blotting paper. Heart weight/tail length ratios are primary indicators of cardiac hypertrophy. ANP, BNP, and *β*-MHC gene expression in the heart were also measured as markers of cardiac hypertrophy.

### 2.6. Preparation of Heart Tissue Homogenate

Rat heart tissue homogenate was prepared by homogenizing 100 mg of heart tissue in 2 mL of 0.05 M phosphate buffer (pH 7.4) and centrifuging at 15,000 rpm for 30 min at 4°C. The resulting supernatant was stored at -80°C for further analysis.

### 2.7. Myocardial Endogenous Antioxidant and Lipid Peroxidation Status

The superoxide dismutase (SOD) activity was determined by using a commercially available assay kit (Sigma-Aldrich) [14]. In this assay, the rate of pyrogallol autoxidation is measured and indicated by a change (increase) in optical density at 420 nm. Myocardial glutathione (GSH) content in heart homogenate was measured by biochemical assay using the dithionitrobenzoic acid (DTNB) method as described earlier [13]. Data were expressed as micrograms per milligram protein. Myocardial catalase (CAT) activity was determined by measuring the decomposition of hydrogen peroxide at 240 nm, according to the method described earlier [15]. Data were expressed as milliunits per microgram protein. The extent of lipid peroxidation in the heart was determined by measuring malondialdehyde (MDA) content according to a modified method based on the reaction with thiobarbituric acid [8]. Data were expressed as micromoles per gram heart weight using the extinction coefficient of 1.56 × 10^−5^ M^−1^ cm^−1^.

### 2.8. Gene Expression Profiling

RNA was isolated from the heart tissue of all groups (*N* = 4) using the TRI Reagent (Sigma-Aldrich) following the manufacturer's protocol. Quantification and quality assessment of RNA was carried out with the NanoDrop Spectrophotometer (Thermo Fisher Scientific) and running on 1% agarose gel prepared in DEPC-treated TBE buffer. The extracted RNA was stored at -80°C for future use. RNA was treated with DNase before cDNA synthesis. cDNA was synthesized using 1 *μ*g RNA with the SuperScript III Reverse Transcriptase (Takara, USA). A reverse transcriptase polymerase chain reaction was carried out using the EmeraldAmp PCR Master Mix and primers for target genes. The data were normalized to the expression of the reference gene, ribosomal protein L32 (RPL32) [16]. The list of primers is provided in [Supplementary-material supplementary-material-1] in the supplementary material.

### 2.9. Immunoblotting

Rat heart tissues were processed using tissue protein extraction according to the manufacturer's protocol (Thermo Fisher Scientific). After centrifugation at 16,000 × g for 5 min, the protein in the supernatant (cytoplasmic extract) was transferred to a prechilled tube. Insoluble fraction (pellet) was suspended in an ice-cold nuclear extraction reagent, vortexed, and centrifuged at 16,000 × g for 10 min. Protein was quantified by the BCA method (Thermo Fisher Scientific). Protein (30 *μ*g) was resolved in 10% SDS-polyacrylamide gel using the TGX Stain-Free™ FastCast™ (Bio-Rad™). After electrophoresis, protein was transferred to a polyvinylidene difluoride membrane (GE Healthcare). Blocking of the membrane was performed using 3% nonfat milk in TBS-T containing 0.1% Tween 20 at room temperature for 1 h, followed by an appropriate primary antibody treatment overnight at 4°C. The membrane was washed with TBS-T for 5 min (three times). After washing, the membrane was incubated with the corresponding HRP-labeled secondary antibody at room temperature for 1 h. The membrane was washed with TBS-T (3 times for 5 min each), and the blot was visualized using the ChemiDoc XRS+ Imager (Bio-Rad) using the Lumi-Light Western Blotting Substrate (Roche). Different antibodies used for the study are SOD2 (Cell Signaling Technology; Cat no. 13141S), caspase-3 (Cell Signaling Technology; Cat no. 9664S), and anti-rabbit and anti-mouse secondary antibodies.

### 2.10. Electrocardiogram Recording

Electrocardiogram (ECG) was recorded on the 13th day of the experiment. Animals were anesthetized using ketamine (75 mg/kg, IP) and xylazine (5 mg/kg, IP) in a mixture and kept in a supine position on a homeothermic blanket to maintain the body temperature throughout the experiment. ECG was measured using the PowerLab with LabChart software as described before (*N* = 4) [17].

### 2.11. Histopathology

Myocardial tissue was fixed in 4% neutral buffered formalin for 48 h. Fixed tissue was processed routinely and embedded in paraffin. Paraffin sections (5 *μ*m) were cut and mounted on glass slides, stained with Hematoxylin and Eosin (H&E) and Masson's trichrome stains, and examined under a light microscope [1].

### 2.12. Cell Culture to Develop In Vitro Model

The H9c2 cell line, derived from embryonic rat heart tissue, was used in the present study to induce cellular hypertrophy [1]. H9c2 cells were purchased from ATCC (USA) and cultured in DMEM containing 10% fetal bovine serum (FBS). Cells from passages 2-7 were used for all the experiments. H9c2 cells were subjected to different treatment protocols described as follows:

- Model 1: Isoproterenol- (ISO-) induced cardiac hypertrophy and inflammation

1. CON: Vehicle
2. ISO: Cells were treated with isoproterenol (50 *μ*M) for 24 h
3. ISO+MB: Cells were treated with isoproterenol (50 *μ*M) for 24 h along with fresh aqueous MB extract obtained from MB powder with 25 *μ*g/mL concentration

(b) Model 2: Lipopolysaccharide- (LPS-) induced inflammation

1. CON: Vehicle
2. LPS: Cells were treated with lipopolysaccharide (1 *μ*g/mL) for 24 h
3. LPS+MB: Cells were treated with lipopolysaccharide (1 *μ*g/mL) for 24 h along with fresh aqueous MB extract obtained from MB powder with 25 *μ*g/mL concentration

### 2.13. Measurement of Intracellular Reactive Oxygen Species (ROS)

To evaluate the antioxidant activity of MB on H9c2 cells against isoprotenol- and LPS-induced oxidative stress, intracellular ROS were measured using 2′,7′-dichlorofluorescin diacetate (DCFH-DA; Invitrogen, Carlsbad, CA, USA) by flow cytometry (BD FACSCanto II, USA) [18]. Briefly, we treated H9c2 cells with isoproterenol (50 *μ*M) and isoproterenol plus MB (25 *μ*g/mL) or chlorogenic acid (CGA) (50 *μ*M), and LPS (1 *μ*g/mL) and LPS plus MB (25 *μ*g/mL) or CGA (50 *μ*M) for 24 hrs, and intracellular ROS levels were measured. After the incubation period, cells were incubated with 10 *μ*M H2DCFDA for 30 min, then cells were harvested and washed with PBS twice and intracellular reactive oxygen species were measured by a flowcytometer by using the FITC channel. We recorded 2 × 10^4^ cells per reaction, and data was analysed using the FlowJo version 10.0.6 software.

### 2.14. Statistical Analysis

Statistical analysis was carried out using the Origin Pro 9.0 software packages (OriginLab Corporation, Northampton, MA, USA). All values were expressed as mean ± SEM. Data from more than two groups were statistically analyzed using one-way ANOVA for multiple group comparison followed by Bonferroni *post hoc* test.

## 3. Results

### 3.1. Identification of Phenolic Compounds from *Musa balbisiana* Powder

The screening and identification of phenolic compounds present in *Musa balbisiana* fruit pulp powder extract were performed by LC-ESI-MS/MS. From metabolomics data, we identified different phenolic compounds present in *Musa balbisiana* (Table 1). The identified different phenolic compounds and their role in different diseases are illustrated in [Supplementary-material supplementary-material-1]. The peak identification was performed by comparison of the retention time (RT), *λ* max, and mass spectra of MB with the standard compounds and earlier literature reports (Table 1). RTs (min) of 2.03, 3.41, 4.75, 8.00, 8.001, 8.001, and 8.11 were identified with the following: chlorogenic acid, (-)-epicatechin, catechol, kaempferol 3-O-sophoroside, quercetin, rutin, and apigenin-6-C-glucoside-7-O-glucoside. (-)-Epicatechin and chlorogenic acid have been verified with the retention time and MS/MS fragmentation pattern (data not shown).

### 3.2. *Musa balbisiana* Treatment Attenuated Cardiac Hypertrophy in Rat Heart

**Figure 1:**
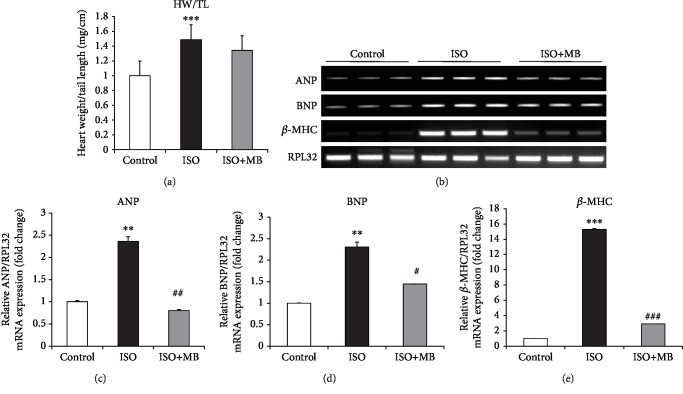
Effect of *Musa balbisiana* on isoproterenol-induced cardiac hypertrophy. (a) Representation of cardiac hypertrophy by heart weight and tail length ratio. (b) Representation of gene expression of ANP, BNP, and *β*-MHC. (c) Expression of ANP mRNA as a marker of cardiac hypertrophy. (d) Expression of BNP mRNA as a marker of cardiac hypertrophy. (e) Expression of *β*-MHC mRNA as a marker of cardiac hypertrophy. Data shown as mean ± SEM, ^∗∗^*p* < 0.01 and ^∗∗∗^*p* < 0.001 vs. CON; ^#^*p* < 0.05, ^##^*p* < 0.01, and ^###^*p* < 0.001 vs. ISO groups.

Heart weight/tail length ratio was evaluated as a measurement of cardiac hypertrophy. A significant increase in heart weight/tail length ratio was observed in the ISO group compared to control group. However, feeding of MB decreased the heart weight/tail length ratio compared to the ISO group (Figure 1(a)). However, this change was not statistically significant. Further, to find the effect of MB on cardiac hypertrophy, ANP, BNP, and *β*-MHC gene expressions were measured as markers of cardiac hypertrophy (Figure 1(b)). ANP and BNP gene expression was significantly (*p* < 0.01) increased in the ISO group compared to the control group and significantly (*p* < 0.05) decreased in the ISO+MB group compared to the ISO group (Figures 1(c) and 1(d)). Similarly, *β*-MHC gene expression was significantly (*p* < 0.001) increased in the ISO group compared to the control group and significantly (*p* < 0.001) decreased by the MB treatment group (ISO+MB) compared to the ISO group (Figure 1(e)).

### 3.3. MB Attenuated Myocardial Fibrosis and Improved Electrocardiograph Parameters in Hypertrophy Heart

**Figure 2:**
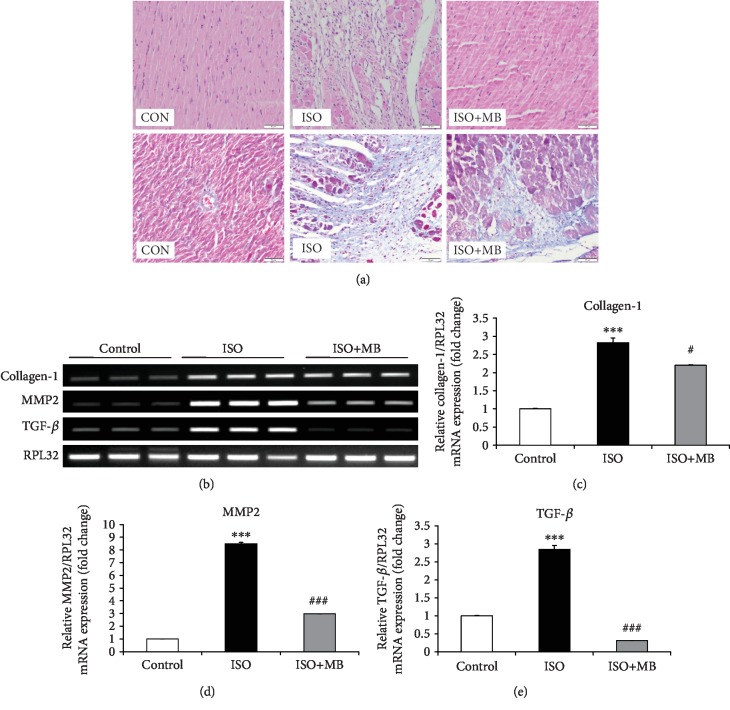
Effect of *Musa balbisiana* on isoproterenol-induced fibrosis and gene expression. (a) Effect of *Musa balbisiana* on isoproterenol-induced histopathological changes in different groups. (b) Representation of gene expression of collagen, MMP2, and TGF-*β*. (c) Collagen mRNA expression. (d) MMP2 mRNA expression. (e) TGF *β* mRNA expression. Data shown as mean ± SEM, ^∗∗∗^*p* < 0.001 vs. CON; ^#^*p* < 0.05 and ^###^*p* < 0.001 vs. ISO groups.

**Figure 3:**
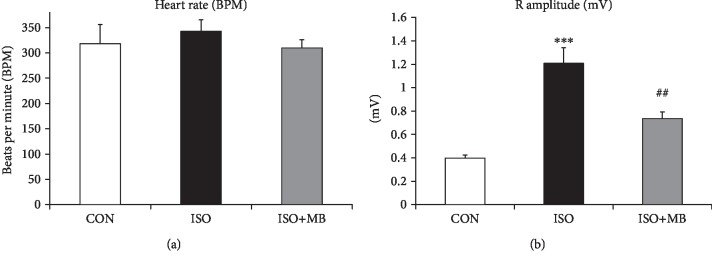
Electrocardiogram (ECG) perturbations in cardiac hypertrophy and effect of *Musa balbisiana*. (a) Heart rate in beats per minute (BPM). (b) *R* amplitude. Data shown as mean ± SEM (*N* = 4), ^∗∗∗^*p* < 0.001 vs. CON; ^##^*p* < 0.01 vs. ISO groups.

We observed large areas of myocardial fibrosis along with leukocyte infiltration in the ISO group after staining with both Masson's trichrome and Hematoxylin and Eosin staining. While light micrograph of the control heart showed normal architecture and no fibrosis, the hypertrophic heart (ISO) showed fibrosis in cardiac muscle along with the infiltration of acute and chronic inflammatory cells. However, fibrosis was reduced with the reduction of myocardial injury in the ISO+MB group compared to the ISO group (Figure 2(a)). Additionally, collagen-1, MMP2, and TGF-*β* mRNA expressions were measured as markers of fibrosis and myocardial remodeling (Figure 2(b)). A significant (*p* < 0.001) increase in the expression of collagen-1 was observed in the ISO group compared to the control group. However, a significant (*p* < 0.05) decrease in the expression of collagen was observed in the ISO+MB group compared to the ISO group (Figure 2(c)). Similarly, MMP2 and TGF-*β* expression was increased significantly (*p* < 0.001) in the ISO group compared to the control group. However, a significant (*p* < 0.001) decrease in the expression of MMP2 and TGF-*β* was observed in the ISO+MB group compared to the ISO group (Figures 2(d) and 2(e)). Electrocardiogram analysis showed increased *R* amplitude along with an increased heart rate (Figure 3) in the ISO group. All these ECG changes in the hypertrophic heart indicate the presence of cardiac ventricular hypertrophy and tachycardia. MB administration in these rats normalized these altered electrocardiac abnormalities.

### 3.4. MB Attenuated Inflammatory and Proapoptotic Gene Expression in Hypertrophy Heart

**Figure 4:**
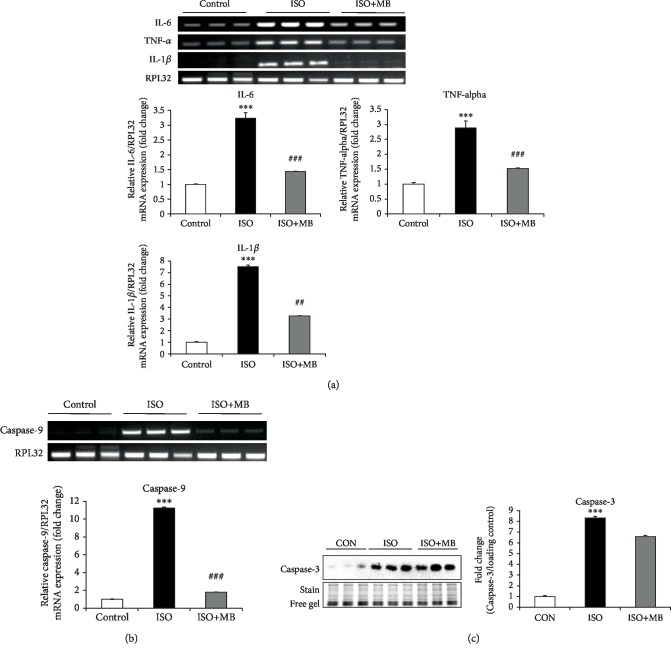
Effect of *Musa balbisiana* on inflammation and apoptosis in hypertrophy heart. (a) Expression of IL-6, TNF-*α*, and IL-1*β* as a marker of inflammation. (b) Expression of caspase-9 mRNA. (c) Protein expression of caspase-3 as a marker of apoptosis. Data shown as mean ± SEM, ^∗^*p* < 0.001 vs. CON; ^##^*p* < 0.01 and ^###^*p* < 0.001 vs. ISO groups.

TNF-*α*, IL-6, and IL-1*β* gene expression was measured as markers of inflammation. Myocardial mRNA expression of three inflammatory genes, i.e., TNF-*α*, IL-6, and IL-1*β* was significantly (*p* < 0.001) increased in the ISO group as compared to the control group (Figure 4(a)). MB decreased the mRNA expression of myocardial TNF-*α* (*p* < 0.001) as compared to the ISO group (Figure 4(a)). Likewise, we also observed a reduction of IL-6 (*p* < 0.001) and IL-1*β* (*p* < 0.01) gene expression by MB treatment compared to the ISO-treated group (Figure 4(a)). In addition, caspase-9 gene and caspase-3 protein expressions were measured as markers of apoptosis. A significant (*p* < 0.001) increase in the expression of these apoptosis markers was observed in the ISO group compared to the control group. A significant (*p* < 0.001) decrease in the expression of the caspase-9 gene was observed in the ISO+MB group compared to the ISO group (Figure 4(b)). MB treatment did not reduce the increased level of caspase-3 in the ISO-treated rat heart (Figure 4(c)).

### 3.5. MB Treatment Attenuated Oxidative Stress in Hypertrophy Rat Heart

**Figure 5:**
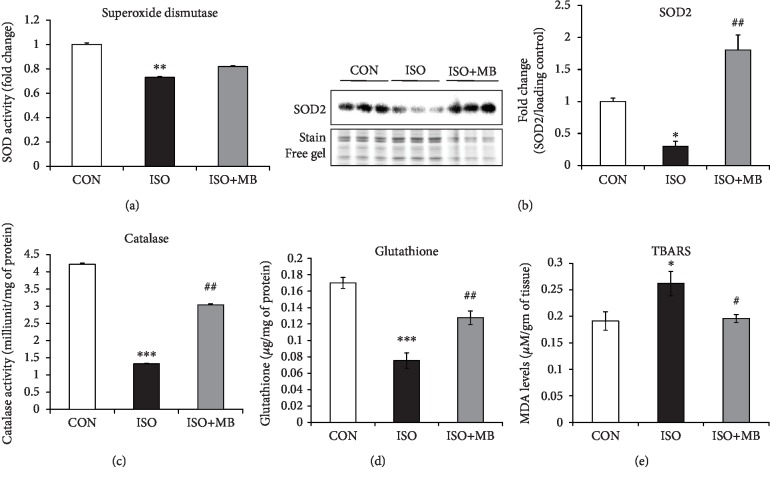
Effect of *Musa balbisiana* on redox status in hypertrophy heart: (a) superoxide dismutase activity, (b) protein expression of MnSOD, (c) catalase activity, (d) glutathione activity, and (e) thiobarbituric acid reactive substances (TBARS). Data shown as mean ± SEM (*N* = 5), ^∗^*p* < 0.05, ^∗∗^*p* < 0.01, and ^∗∗∗^*p* < 0.001 vs. CON; ^#^*p* < 0.05 and ^##^*p* < 0.01 vs. ISO groups.

To analyze the oxidative stress in hearts from all groups, we measured myocardial endogenous antioxidants like SOD, catalase and reduced glutathione (GSH) levels and myocardial lipid peroxidation (Figure 5). Myocardial MnSOD protein expression was significantly decreased in ISO animals (*p* < 0.05). However, MB treatment restored the decreased level of MnSOD (*p* < 0.01) (Figure 5(b)). There was a significant decrease in myocardial catalase (*p* < 0.001) and SOD (*p* < 0.01) activity in the ISO group compared to the control group. However, an increase in myocardial SOD and catalase activity was observed in the ISO+MB group compared to the ISO group (Figures 5(a) and 5(c)). Likewise, a significant (*p* < 0.001) decrease in myocardial GSH level was observed in the ISO group compared to the control group. However, there was a significant (*p* < 0.01) increase in GSH level observed in the ISO+MB group compared to the ISO group (Figure 5(d)). A significant (*p* < 0.05) increase in myocardial TBARS level was observed in the ISO group compared to the control group, indicating lipid peroxidation. However, there was a significant (*p* < 0.05) decrease in myocardial TBARS level in the ISO+MB group compared to the ISO group (Figure 5(e)).

### 3.6. MB Attenuated Hypertrophy and Inflammatory Gene Expression in H9c2 Cells

**Figure 6:**
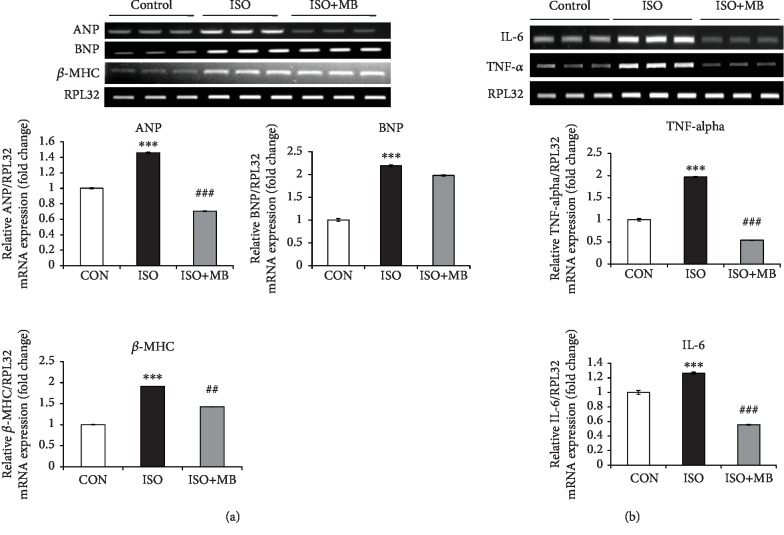
Effect of *Musa balbisiana* against isoproterenol-treated H9c2 cells. (a) Expression of ANP, BNP, and *β*-MHC mRNA as a marker of cardiac hypertrophy. (b) Expression of IL-6 and TNF-*α* mRNA as a marker of inflammation. Data shown as mean ± SEM, ^∗∗∗^*p* < 0.01 vs. CON; ^##^*p* < 0.01 and ^###^*p* < 0.001 vs. ISO groups.

The effect of the water extract of MB was evaluated on H9c2 cells against isoprotenol-induced hypertrophy and LPS-induced inflammation. ANP and BNP gene expression was significantly (*p* < 0.001) increased in the ISO group compared to the control group. A significant (*p* < 0.001) decrease in ANP expression was observed only in the ISO+MB group compared to the ISO group. However, BNP expression was not found significant. Similarly, *β*-MHC gene expression was significantly (*p* < 0.001) increased in the ISO group compared to the control group and significantly (*p* < 0.01) decreased in the MB treatment group (ISO+MB) compared to the ISO group (Figure 6(a)). In addition, myocardial mRNA expression of inflammatory genes, i.e., IL-6 and TNF-*α* was significantly (*p* < 0.001) increased in the ISO group as compared to the control group. However, a significant (*p* < 0.001) decrease of both gene expressions was observed after MB treatment (Figure 6(b)).

**Figure 7:**
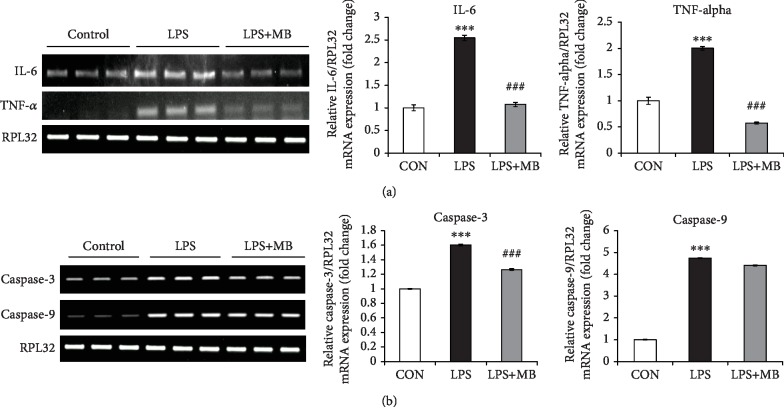
Effect of *Musa balbisiana* against LPS-treated H9C2 cells. (a) Expression of IL-6 and TNF-*α* mRNA as a marker of inflammation. (b) Expression of caspase-3 and caspase-9 mRNA as a marker of apoptosis. Data shown as mean ± SEM, ^∗∗∗^*p* < 0.001 vs. CON; ^###^*p* < 0.001 vs. LPS groups.

In the lipopolysaccharide (LPS)- induced inflammation model, IL-6 and TNF-*α* were significantly (*p* < 0.001) increased in the LPS group as compared to the control group (Figure 7(a)). We have also observed that the levels of IL-6 and TNF-*α* were significantly (*p* < 0.001) decreased in the MB treatment group as compared to the LPS-treated group. Likewise, caspase-3 and caspase-9 expression was measured as markers of apoptosis. A significant (*p* < 0.001) increase in the expression of these genes was observed in the LPS group compared to the control group. However, a decreased expression of these genes was observed in the LPS+MB group compared to the LPS group (Figure 7(b)).

### 3.7. MB Attenuated Isoproterenol- and Lipopolysaccharide-Induced ROS Generation in H9c2 Cells

**Figure 8:**
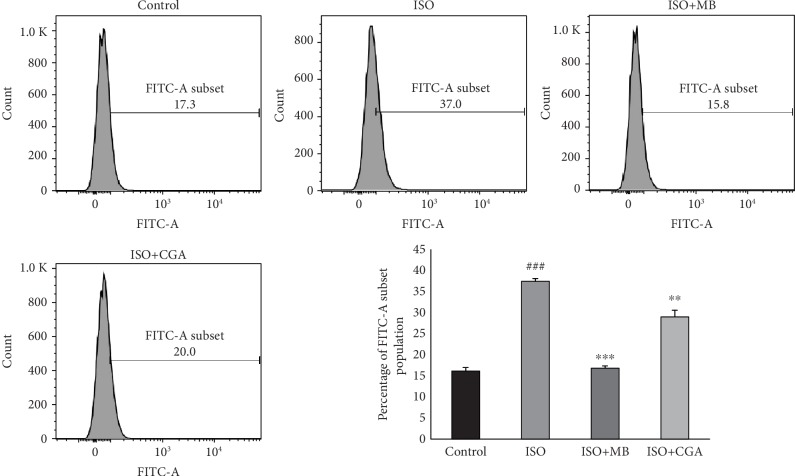
Antioxidant activity of *Musa balbisiana* (25 *μ*g/mL) and chlorogenic acid (50 *μ*M) on isoproterenol- (50 *μ*M) induced oxidative stress in H9c2 cells.

**Figure 9:**
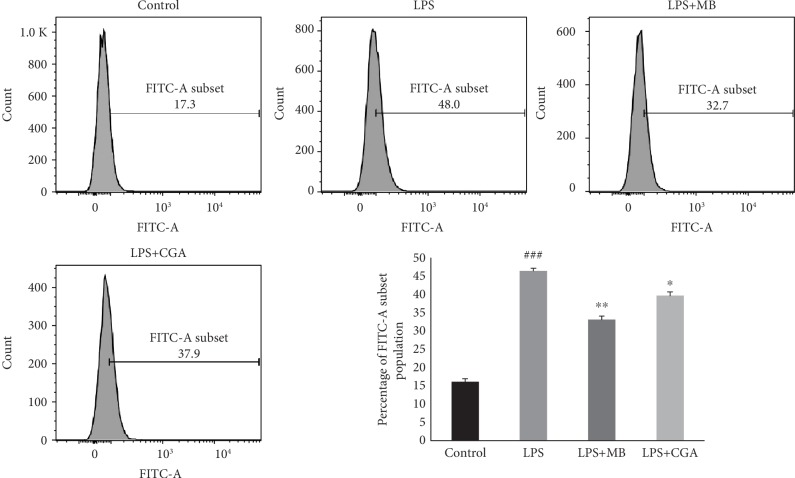
Antioxidant activity of *Musa balbisiana* (25 *μ*g/mL) and chlorogenic acid (50 *μ*M) on LPS- (1 *μ*g/mL) induced oxidative stress in H9c2 cells.

We studied the effect of the water extract of MB powder on isoproterenol- and LPS-induced ROS generation in H9c2 cells and compared effect with chlorogenic acid, the phenolic compound present in MB fruit powder (Figures 8 and 9). There was 2-fold and 3-fold increase in intracellular reactive oxygen species (ROS) after isoproterenol and LPS treatment in H9c2 cells, respectively, as compared to control H9c2 cells. Further, we found a significant decrease in intracellular ROS generation in isoproterenol-treated H9c2 cells in the presence of MB and chlorogenic acid. Similarly, there was a significant decrease in intracellular ROS generation in LPS-treated H9c2 cells in the presence of MB and chlorogenic acid. In both *in vitro* models, the MB water extract showed better efficacy than chlorogenic acid.

## 4. Discussion

Plants rich in polyphenolic compounds have been reported to play a key role in reducing the progression of chronic diseases such as cardiovascular diseases, cancer, tumorigenesis, diabetes, and chronic inflammatory disorders [8, 9]. Among the chronic diseases, cardiovascular disease represents the highest in terms of global deaths. There is a huge global demand for safe and effective natural nutraceutical agents that can provide preventive health benefits. Therefore, there is a need to search for novel cardioprotective agents with lesser side effects. Folklore studies have revealed that among the different varieties of *Musa* sp., *Musa balbisiana* Colla has a high nutritional value. Recent data also reported its effects in the treatment of diabetes and hyperlipidemia [5]. Here, we validate the traditional use of *Musa balbisiana* fruit pulp powder that may improve cardiovascular responses in a rat model of isoproterenol- (ISO-) induced cardiac hypertrophy. To chemically characterise *Musa balbisiana* fruit powder, we did LC-MS analysis. The present study confirmed the presence of several phenolic compounds like catechol, epicatechin, chlorogenic acid (CAA), quercetin, rutin, and kaempferol in *Musa balbisiana* fruit powder. A growing number of scientific reports show that high polyphenol intake from fruits and vegetables is associated with a decreased risk of many human diseases such cardiovascular and degenerative diseases [9, 19–21]. Intake of chlorogenic acid, quercetin and apigenin, present in *Musa balbisiana* fruit powder, have been associated with a decreased risk of cardiovascular disease and type 2 diabetes [11, 22–24]. Recently, it was reported that the beneficial effects of phenolic compounds have been attributed to their anti-inflammatory and antioxidant properties [25–27]. Earlier reports also demonstrate several plant extracts showing their beneficial effect through strong antioxidant properties, which are mainly due to the presence of polyphenol compounds [28–30]. In the present study, we evaluated the potential therapeutic effect of *Musa balbisiana* fruit pulp powder, rich in polyphenols, on isoproterenol-induced cardiac hypertrophy and explored the possible mechanisms involved. Earlier reports also demonstrated the beneficial effect of root extract of *M. balbisiana* in reducing hyperlipidemia and diabetes [5]. However, chemical characterization of the *Musa balbisiana* fruit, as well as the role of *Musa balbisiana* to reduce cardiac hypertrophy and its associated inflammation, was not observed yet.

Isoproterenol enhances the atrial natriuretic peptide (ANP) and brain natriuretic peptide (BNP) and accelerates cardiac hypertrophy as reported by previous studies [1, 13, 16, 31]. In the present study, MB decreased cardiac hypertrophy as observed by a reduction slight of heart weight/tail length ratio and expression of fetal genes such as ANP, BNP, and *β*-MHC. Similarly, previous studies have shown that ANP and BNP are secreted in large amounts when cardiac hypertrophy occurs [1, 31]. Increased myocardial fibrosis and collagen deposition are hallmarks of cardiac hypertrophy [13, 32, 33]. In the present study, histopathological examination of the heart tissue showed fibrosis in cardiac muscle along with the infiltration of acute and chronic inflammatory cells in the ISO group. Our finding showed that fibrosis was reduced and myocyte injury was decreased in the ISO+MB group compared to the ISO group (Figure 2(a)). In addition, genes involved in cardiac fibrosis such as collagen-1, MMPs, and TGF-*β* level were overexpressed in hypertrophy, which is quite in agreement with previous studies [16, 31]. MB significantly reduced fibrosis and the expression of marker genes like collagen, MMPs, and TGF-*β*. The present study also demonstrated that MB improved cardiac electrophysiology as observed in electrocardiogram (ECG) analysis. ISO administration in rats increased the heart rate as well as the *R* wave amplitude, which are the indicators of left ventricular hypertrophy [17]. MB showed a improvement in both electrophysiology parameters.

In the animal model of cardiac hypertrophy, we have observed increased inflammation. Recent literature shows that damage-associated molecular patterns (DAMPs) released from the diseased heart tissue alone are sufficient to induce chronic myocardial inflammation. Cardiac hypertrophy is well-known to enhance DAMPs and thus responsible for inflammatory gene expression. In the present study, we have observed enhanced expression of inflammatory genes such as TNF-*α*, IL-6, and IL-1*β* in hypertrophy heart. Administration of MB decreased the expression of these three genes when compared to the hypertrophy group. Many research studies have demonstrated that chronic inflammation in hypertrophy heart is associated with ROS generation and may be responsible for cardiac remodeling [11, 31]. ROS trigger a large variety of hypertrophy signaling transcriptional factors and reduce endogenous antioxidants [31, 34, 35]. To analyze the cardiac redox status in all groups, we have measured endogenous antioxidants like SOD, catalase and reduced glutathione (GSH) levels and lipid peroxidation like TBARS. In the present study, we found that isoproterenol decreased SOD, GSH, and catalase, and increased lipid peroxidation products such as TBARS in hypertrophic heart. Oral administration of MB fruit powder normalized endogenous antioxidant levels and TBARS in hypertrophy heart. Thus, MB treatment in ISO animals prevented these detrimental perturbations through the attenuation of inflammation and oxidative stress. In the heart, SOD is the primary defense against oxidative stress [36]. SOD2, a mitochondrial SOD, plays an important role in regulating mitochondrial and cellular ROS level. In the present study, the level of MnSOD in hypertrophy heart is significantly decreased. However, MB treatment significantly restored the decreased level of MnSOD in hypertrophy heart. In addition, the levels of caspase-3 and caspase-9, which contribute to cardiac cell apoptosis in failing hearts, were significantly increased in ISO heart. However, MB treatment decreased the level of caspase-9, but not caspase-3 [37].

To confirm our *in-vivo* data, we have also treated rat ventricular cardiomyoblasts, H9c2 cells with isoproterenol in presence of *Musa balbisiana*. Our data confirmed that increased expression of ANP, BNP, and *β*-MHC in isoproterenol-treated H9c2 cells was significantly decreased after MB treatment. Similarly, increased inflammatory markers such as IL-6 and TNF-*α* mRNA levels were significantly decreased after MB treatment. This *in vitro* experiment confirmed our hypothesis that MB has antihypertrophic as well as anti-inflammatory properties. To confirm the inflammatory effect, we have developed another model of inflammation after treating H9c2 cells with lipopolysaccharide (LPS). Increased inflammatory markers such as IL-6 and TNF-*α* mRNA levels were observed in LPS-treated H9c2 cells but were significantly decreased after MB treatment. Further, to confirm our *in vivo* data, we have also observed the effect of MB on isoproterenol- and LPS-induced ROS generation in H9c2 cells. The effect of MB was compared with a standard antioxidant polyphenolic compound, chlorogenic acid, present in MB powder. Data showed that water extract from MB powder showed a higher antioxidant property as compared to the chlorogenic acid. These data confirm that the presence of chlorogenic acid along with other polyphenolic compounds in MB powder is responsible for the beneficial effect. Our LC/MS study has identified several polyphenols like catechol, (-)-epicatechin, kaempferol 3-O-sophoroside, quercetin 3-O-[2^″^-O-b-D-glucopyranosyl]-a-L-rhamnopyranoside, rutin, apigenin-6-C-glucoside-7-O-glucoside, and chlorogenic acid. The presence of all these polyphenols in MB fruit powder might be responsible for the attenuation of cardiac hypertrophy through modulating inflammation and oxidative stress.

## 5. Conclusion

In conclusion, the present data proved that dried *Musa balbisiana* fruit pulp powder is one of the promising nutritional candidates for the prevention of cardiac hypertrophy through modulating inflammation and oxidative stress in hypertrophy heart. Additionally, the results of the present study also indicate the presence of several phenolic antioxidants such as catechol, (-)-epicatechin, kaempferol 3-O-sophoroside, quercetin 3-O-[2^″^-O-b-D-glucopyranosyl]-a-L-rhamnopyranoside, rutin, apigenin-6-C-glucoside-7-O-glucoside, and chlorogenic acid in *Musa balbisiana*, which might be responsible for its beneficial effect to improve cardiac health.

## Figures and Tables

**Table 1:** Peak identification (LC/MS data) from *Musa balbisiana* fruit pulp powder extracts and confirmation of phenolic compounds using analytical standards.

S. no.	Phenolic compounds	Retention time (min)	Empirical formula	*m*/*z*	Adducts	Fragmentation score
1.	Chlorogenic acid	2.03	C_16_H_18_O_9_	377.0849	M+H	0.0127
2.	(-)-Epicatechin	3.41	C_15_H_14_O_6_	289.0714	M-H	75.8
3.	Catechol	4.75	C_6_H_6_O_2_	109.0290	M-H	81.9
4.	Kaempferol 3-O-sophoroside	8.00	C_27_H_30_O_16_	609.1454	M-H	16.3
5.	Quercetin 3-O-[2^″^-O-b-D-glucopyranosyl]-a-L-rhamnopyranoside	8.001	C_27_H_30_O_16_	609.1454	M-H	80
6.	Rutin	8.001	C_27_H_30_O_16_	609.1454	M-H	76.2
7.	Apigenin-6-C-glucoside-7-O-glucoside	8.11	C_27_H_30_O_15_	639.1560	M+FA-H	0.0614

## Data Availability

The data used to support the findings of this study are available from the corresponding author upon request.

## Abbreviations

MB: *Musa balbisiana*
ISO: Isoproterenol
ANP: Atrial natriuretic peptide
*β*-MHC: Beta myosin heavy chain
TGF-beta: Transforming growth factor-beta
TNF-*α*: Tumor necrosis factor alpha
UHPLC: Ultra-high-performance liquid chromatography
CGA: Chlorogenic acid
MMP2: Matrix metalloproteinase 2
TGF-*β*: Transforming growth factor-beta
SD: Sprague-Dawley
SOD2: Superoxide dismutase 2
ANOVA: Analysis of variance.

## Supplementary Materials

Supplementary Materials Table S1: primer sequences. Table S2: phytochemicals present in *Musa balbisiana*.
