# Supplementary material for: Musa balbisiana Fruit Rich in Polyphenols Attenuates Isoproterenol-Induced Cardiac Hypertrophy in Rats via Inhibition of Inflammation and Oxidative Stress
Source: Oxid Med Cell Longev. 2020 Jan 27;2020:7147498. doi: 10.1155/2020/7147498 (PMC7007945; doi:10.1155/2020/7147498)
Supplement: Supplementary Materials — Table S1: primer sequences. Table S2: phytochemicals present in Musa balbisiana. [file 7147498.f1.pdf]

Supporting Information

(Table S1: List of primers, Table S2: List of phenolic compounds present in *Musa balbisiana*)

Musa balbisiana fruit rich in polyphenols attenuates isoproterenol-induced cardiac hypertrophy in rats via inhibition of inflammation and oxidative stress

Sima Kumari[1], Parmeshwar B. Katare[1], R. Elancheran[2], Hina L. Nizami[1], Bugga Paramesha[1], Sudheer Arava[3], Partha Pratim Sarma[4], Roshan Kumar[1], Dinesh Mahajan[1], Yashwant Kumar[1], Rajlakshmi Devi[4], Sanjay K. Banerjee[1*]

[1]Drug Discovery Research Center (DDRC), Translational Health Science and Technology Institute (THSTI), Faridabad, India
[2]Drug Discovery Lab, Department of Chemistry, Annamalai University, Tamil Nadu
[3]Department of Pathology, All India Institute of Medical Sciences (AIIMS), New Delhi, India
[4]Institute of Advanced Study in Science and Technology, Guwahati-781035, Assam, India

Legends:

Table S1 Primers sequences

Table S2 Phytochemicals present in *Musa balbisiana*

Materials and Methods

Gene expression profiling

Table S1: Primers sequences

Name	Sequences	Product size (bp)	Temperature
RPL32	AGATTCAAGGGCCAGATCCT<br>CGATGGCTTTTCGGTTCTTA	193	57.3<br>55.3
TNFα	CGACTCTGACCCCCATTACT<br>CGTCTCGTGTGTTTCTGAGC	153	59.4<br>59.4
ANP	AGCGAGCAGACCGATGAAG<br>AGCCCTCAGTTTGCTTTTCA	146	57.3<br>55
BNP	GACCAAGGCCCTACAAAAGA<br>CCCAAAGCAGCTTGAACTATG	102	54.8<br>54.2
βMHC	TGGAGCTGATGCACCTGTAG<br>ACTTCGTCTCATTGGGGATG	164	56.8<br>54.5
Caspase 3	AGGCCGACTTCCTGTATGCT<br>TCCGGTTAACACGAGTGAGG	155	59.4<br>59.4
Caspase 9	AAGACCATGGCTTTGAGGTG<br>CAGGAACCGCTCTTCTTGTC	210	57.3<br>59.4
Collagen 1	ACGTCCTGGTGAAGTTGGTC<br>CAGGGAAGCCTCTTTCTCCT	175	59.4<br>59.4
Mmp2	GGGTGGTGGTCACAGCTATT<br>CGGTGTGCAGTGAAGATTGT	156	57.2<br>55.7
Mmp9	CCACCGAGCTATCCACTCAT<br>GTCCGGTTTCAGCATGTTTT	159	56.4<br>53.9

Results

Identification of phenolic compounds from *Musa balbisiana* powder

Table S2: Phytochemicals present in *Musa balbisiana*

Name of the Compound	Empirical formula	Retention time (min)	Ethno medical uses	Sources
Catechol	$C_6H_6O_2$	4.753666667	Cardioprotective and Antilipidemic properties	[21, 22]
Rutin	$C_{15}H_{14}O_6$	8.001	Cardioprotective effects and antioxidant activity	[27, 28]
(-)-Epicatechin	$C_{27}H_{30}O_{16}$	3.41	Antioxidant activity and cardioprotective effects	[29, 30]
Kaempferol 3-O-sophoroside	$C_{27}H_{30}O_{16}$	8.001416667	Cardioprotective, antimicrobial and antioxidant activity	[31, 32]
Quercetin-3,4'-O-di-beta-glucopyranoside	$C_{27}H_{30}O_{16}$	8.001416667	Cardioprotective effects and antioxidant activity	[10, 23, 24]
Apigenin-6-C-glucoside-7-O-glucoside	$C_{27}H_{30}O_{15}$	8.1183	Cardioprotective effects and antioxidant, antidiabetic activity	[10, 25, 26]
Chlorogenic acid	$C_{16}H_{18}O_9$	2.03	Anti-inflammatory, antioxidant and Cardioprotective	[10, 20]
